# Scientific Productions on Precision Livestock Farming: An Overview of the Evolution and Current State of Research Based on a Bibliometric Analysis

**DOI:** 10.3390/ani13142280

**Published:** 2023-07-12

**Authors:** Rosanna Marino, Francesca Petrera, Fabio Abeni

**Affiliations:** Centro di Ricerca Zootecnia e Acquacoltura, Consiglio per la Ricerca in Agricoltura e l’Analisi dell’Economia Agraria (CREA), Via Lombardo 11, 26900 Lodi, Italy; francesca.petrera@crea.gov.it (F.P.); fabiopalmiro.abeni@crea.gov.it (F.A.)

**Keywords:** precision livestock farming, smart farming, bibliometric analysis, bibliometrix R

## Abstract

**Simple Summary:**

Precision livestock farming (PLF) is a concept that has gained significant interest in recent years due to its potential role in developing sustainable livestock production systems. This study aimed to conduct a bibliometric analysis of the PLF literature from 2005 to 2021 to understand the historical routes that influenced the use of technology in livestock farming, highlight future global trends, and identify changes in scientific research on this topic. The analysis of the PLF literature showed that the number of publications has steadily increased over time, with a more than doubled annual growth rate in the last five years. The leading countries in PLF research were the USA, the Netherlands, and Italy, and the top three core journals publishing PLF research were the *Journal of Dairy Science*, *Computers and Electronics in Agriculture*, and *Animals*. The study also highlighted the central role of automated milking systems in driving innovations in the PLF sector and the growing interest in emissions and mitigation as emerging themes for the future. Overall, this study provides insights into the past, present, and future of PLF research.

**Abstract:**

The interest in precision livestock farming (PLF)—a concept discussed for the first time in the early 2000s—has advanced considerably in recent years due to its important role in the development of sustainable livestock production systems. However, a comprehensive bibliometric analysis of the PLF literature is lacking. To address this gap, this study analyzed documents published from 2005 to 2021, aiming to understand the historical influences on technology adoption in livestock farming, identify future global trends, and examine shifts in scientific research on this topic. By using specific search terms in the Web of Science Core Collection, 886 publications were identified and analyzed using the bibliometrix R-package. The analysis revealed that the collection consisted mostly of research articles (74.6%) and reviews (10.4%). The top three core journals were the *Journal of Dairy Science*, *Computers and Electronics in Agriculture*, and *Animals*. Over time, the number of publications has steadily increased, with a higher growth rate in the last five years (29.0%) compared to the initial period (13.7%). Authors and institutions from multiple countries have contributed to the literature, with the USA, the Netherlands, and Italy leading in terms of publication numbers. The analysis also highlighted the growing interest in bovine production systems, emphasizing the importance of behavioral studies in PLF tool development. Automated milking systems were identified as central drivers of innovation in the PLF sector. Emerging themes for the future included “emissions” and “mitigation”, indicating a focus on environmental concerns.

## 1. Introduction

The quantitative analysis of academic literature is a valuable tool for organizing available knowledge within a topic or specific scientific discipline and identifying the key trends in the field. Interest in precision livestock farming (PLF) has grown in recent years due to its crucial role in developing sustainable livestock production systems. PLF—a concept first discussed in the early 2000s at the 1st European Conference on Precision Livestock Farming held in Berlin in parallel with the 4th European Conference on Precision Agriculture [1]—has evolved through the development of various technologies, starting from transponders for animal identification [2,3], robotic milking [4], and automated feeding, to present-day expectations of the complete automation of farm processes as artificial intelligence and machine learning become more common and readily available [5].

In this story, the introduction of technologies based on a variety of sensors useful for managing a large number of animals, monitoring single animals and the environment, followed more or less the same trend as herd size, which has steadily increased in the last 20 years. In medium and large farms, it is now quite common to have at least one, but usually more than one, PLF technology [6,7,8]. This transformation of livestock farms has proceeded hand in hand with technological developments and the acquisition of advanced systems capable of measuring, collecting, and processing data and transforming them into alerts or feedback for more efficient farm management [9]. However, this evolution/revolution could not (and cannot) only involve the purchase and introduction of new devices or software; it also requires a complete revision of the farm management and organizational model. While several reviews, as reported in the paper, have been written on the topic to clarify the rationale and the application of PLF technologies and how they are becoming common in modern farms, a bibliometric analysis of the peer-reviewed scientific literature has not yet been performed and can provide useful insights [10].

A bibliometric analysis of PLF may be useful to understand: (a) which are the leading sectors in livestock farming for this kind of innovation; (b) which are the technical areas (welfare, reproduction, production, feeding, etc.) expressing the greatest demand for this new approach with digitalization and sensor system implementation; (c) which countries are leading the research and where the main networks of collaboration have formed; and (d) in light of the previous points, what further steps are required in research, but also in agricultural policy and education, to support the long wave of this innovation.

In recent years, bibliometric studies have gained increasing attention in many disciplines [11], and the availability of platforms, such as Scopus and Web of Science, providing reference and citation data for journals, conference proceedings, and other types of documents, along with the development of bibliometric software and packages, such as bibliometrix for R [12], have made it easier to acquire bibliometric datasets from the web and calculate statistics and indexes to paint a picture of a topic’s state of the art, leaving to the “analyser” (the review’s author) the responsibility to interpret the meaning of the results.

Therefore, the purpose of this review is to provide a quantitative analysis of the bibliographic data on articles pertaining to PLF over time. This analysis will highlight the global trends in the PLF literature and provide a comprehensive overview of the present state of scientific and academic research in this crucial and innovative field, with a focus on key contributors (such as authors, institutions, and countries), article sources, and covered topics (documents). Moreover, we intend to identify collaboration networks and emerging research directions, as well as potential future scenarios, limitations, and implementations of the research topic.

## 2. Materials and Methods

On 18 January 2022, we conducted a bibliometric study of documents related to PLF published between 2005 and 2021 using the Web of Science Core Collection database (https://www.webofscience.com, accessed on 18 January 2022). We searched for documents using the topic field with the following keywords: “precision livestock farm*” OR “precision livestock management” OR “precision fish* farm*” OR “automat* milk* system*”. This allowed us to identify papers indexed with the searched words in the title, abstract, authors’ keywords, and Keywords Plus. We used the asterisk as a wild card and quotation marks to limit the search to exact phrases. We then performed data cleaning of the bibliometric data output by first sorting the data by relevance and then inspecting them for thematic pertinence. We exported the list of references as a plain text file formatted to contain the full records (authors, types of documents, subject areas, countries, institutions, journals, funding agencies, citations, and cited references) for each manuscript. The data were then uploaded and converted into a bibliometrix data frame, which was analyzed using Bibliometrix, an R package that allows for both bibliometric and scientometric research. We analyzed the data frame using the biblioshiny web app (version 3.1.4; https://www.bibliometrix.org/home/, accessed on 10 February 2022) [12]. The “summary” function displayed the main information about the bibliographic data frame and several tables, such as annual scientific production, top manuscripts per number of citations, the most productive authors, the most productive countries, total citations per country, the most relevant sources (journals), and the most relevant keywords. As a first step, we performed a descriptive analysis of the data frame, considering three levels of analysis: sources, authors, and documents, to identify the most relevant ones objectively. We then carried out an analysis of the conceptual, intellectual, and social structures of the PLF research field. The conceptual structures were focused on the main themes and trends, using the most important words (author’s keywords or Keywords Plus) in documents (co-occurrence networks and thematic maps). The intellectual structures allowed us to show how specific papers influenced the research trends in the field (co-citation networks, reference publication year spectroscopy, and historiography). The social structure highlighted the collaborations between authors and countries. We used biblioshiny to build the network maps for co-citation, collaboration, and co-occurrence analyses, downloaded pajek files, and used them as inputs for VosViewer, in which networks were normalized via the association strength function and visualized [13,14].

## 3. Results and Discussion

### 3.1. Descriptive Analysis

#### 3.1.1. Main Information and Publication Growth

The WoS search returned a total of 373 documents for the keywords “precision livestock farm*” OR “precision livestock management*”, 9 documents for “precision fish* farm*”, and 524 documents for “automat* milk* system*”. Combining the documents from multiple sources, a total of 886 publications were found and included in the final analysis. It is important to note that some papers were redundant, appearing in more than one set of search results. Therefore, these redundant documents were added to the final dataset only once, avoiding duplication.

The list of these documents, complete with all metadata, is reported in Table S1. Although the search in the WoS Core Collection was feasible from 2005, the retrieved documents only covered the period from 2008. Therefore, the study period reported in the tables and figures was from 2008 up to 2021, encompassing 13 years of PLF-related literature production.

**Figure 1:**
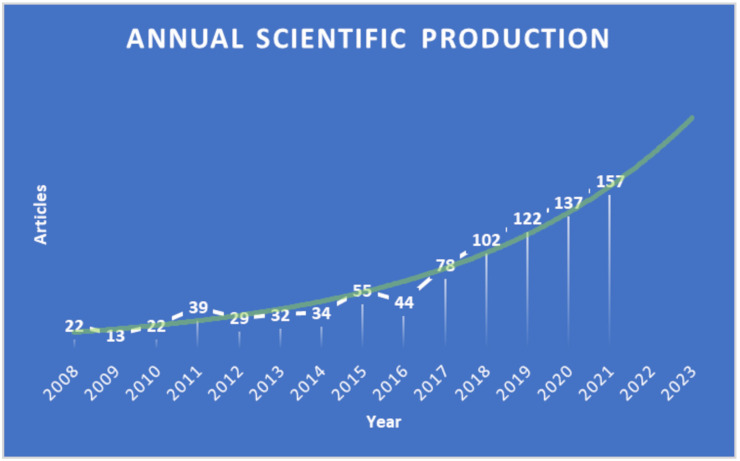
Number of PLF-related documents published per year from 2008 to 2021 (white line) and retrieved from the WoS Core Collection. PLF topic trend determined by fitting document data in an exponential growth model (green line) from 2008 to 2023.

In Table S2, we present a summary of the main information regarding the 886 documents in our collection, of which 665 were research articles and 92 were reviews. Figure 1 illustrates the evolution of the number of articles published during the reported period, with an annual growth rate of 16.3%.

The year with the highest number of published documents was 2021, followed by 2020, while the year with the minimum number of documents was 2009, as shown in Figure 1.

Although the data fitting indicated that the number of documents published per year followed an exponential growth model, the growth rate of publications can be divided into two main periods (Figure 1): the first period (2008–2015) was characterized by a slow but constant increase in the number of publications (annual growth rate of 13.7%), and in the second phase (2016–2021) PLF-related publications showed a considerable and sudden increase (with an annual growth rate of 29.0%).

The number of articles published during the second period comprised about 70% of the total collection (640 documents). The strong and continuous growth, especially after 2016, likely reflects the increasing interest of the scientific community in the use of technologies in modern livestock farming. This finding might be supported by the development and application of new technologies and the opportunities to use them in agriculture, including the management of livestock farms [9,10]. The earliest indexed documents in our collection were all related to automatic milking systems (AMSs) [15,16,17].

#### 3.1.2. Collection Sources

An analysis of the sources of PLF-related documents revealed a total of 222 unique journal titles (Table S2). The *Journal of Dairy Science* emerged as the most significant source, being the leading general dairy research journal worldwide, according to its website (Home Page: *Journal of Dairy Science*). The ten most common journals focused mainly on animal science, animal production, animal welfare, and life sciences, while others were technology-oriented, such as *Computers and Electronics in Agriculture*, *Biosystems Engineering*, and *Sensors* (Table 1). Please refer to Supplementary Table S3 for a complete list.

**Figure 2:**
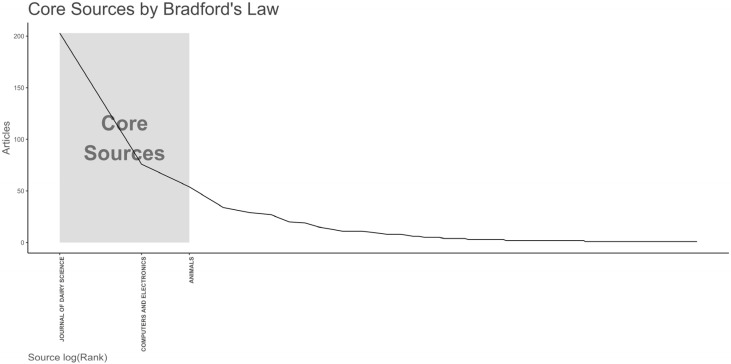
The core journals of PLF-related papers, organized by Bradford’s law.

The *Journal of Dairy Science* was also the most-cited journal in the collection, followed by *Computers and Electronics in Agriculture* and *Applied Animal Behaviour Science* (Table S4). According to Bradford’s law [18], the core sources analysis showed the journals that attracted the highest number of publications on the topic, namely, the *Journal of Dairy Science* (which covered a share of 22.9% of the papers within the studied database), *Computers and Electronics in Agriculture*, and *Animals* (Figure 2).

Our results indicate that the aims, scope, and subject matter are central factors in choosing the appropriate platform for disseminating scientific findings in PLF research. Nevertheless, the quality and reputation of journals also hold significance. Specifically, most PLF-related publications have been featured in three journals with high impact factors: the *Journal of Dairy Science* (IF: 4.225), *Computers and Electronics in Agriculture* (IF: 6.757), and *Animals* (IF: 3.231).

**Figure 3:**
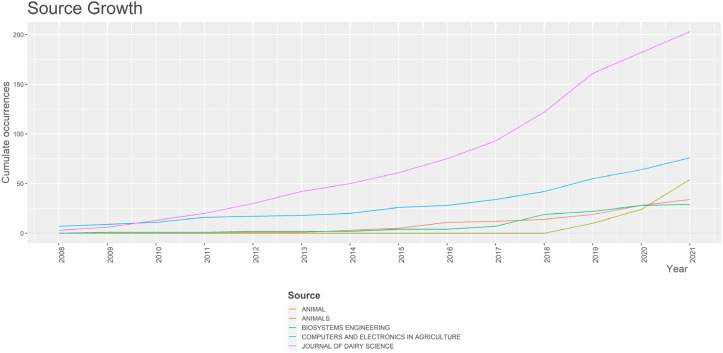
Source dynamics analysis of PLF-related publications, showing the evolution of primary sources over time based on the number of publications on the topic.

However, the *Journal of Dairy Science* and *Computers and Electronics in Agriculture* were the primary sources of PLF papers from the first period of publications in the sector, while *Animals* only emerged as a leading source in 2018, as shown in Figure 3. According to the source dynamics analysis, which tracks the evolution of sources based on the number of publications on the topic (Figure 3), the most productive journals can be divided into two types:

- Journals that began publishing papers on PLF in the early years of the collection and maintained a steady growth throughout the entire period studied, such as the *Journal of Dairy Science* and *Computers and Electronics in Agriculture*;
- Journals that started producing papers in the middle of the period considered (around 2014–2018) and subsequently showed significant and continuous growth, such as *Animal*, *Biosystems Engineering*, and *Animals*.

#### 3.1.3. Relevant Authors and Top PLF Papers

Overall, 2422 authors published documents related to the PLF sector between 2008 and 2021, according to Table S2. On average, each document was written by more than two authors, and each author produced less than one document. In fact, the majority of documents were multi-authored (95%), while only 41 were single-authored. The most relevant authors, based on their publications in the sector, their productivity over time, and their local author impact, are reported in the Supplementary Materials (Table S5 and Figures S1 and S2). In particular, the top ten leading authors contributed to 276 PLF-related papers, mostly multi-authored, which corresponds to 62 fractionalized documents. Table 2A,B provide a list of the top 20 papers with the highest number of citations globally in the entire WOS database or locally based on documents included in the analyzed collection. These papers mainly consist of literature reviews on PLF. The most globally cited paper among them is “Invited review: Changes in the dairy industry affecting dairy cattle health and welfare” [19], with 258 global citations. On the other hand, the most locally cited paper is “Invited review: The impact of automatic milking systems on dairy cow management, behavior, health, and welfare” [20].

#### 3.1.4. Cited References, Reference Publication Year Spectroscopy, and Historiography

**Figure 4:**
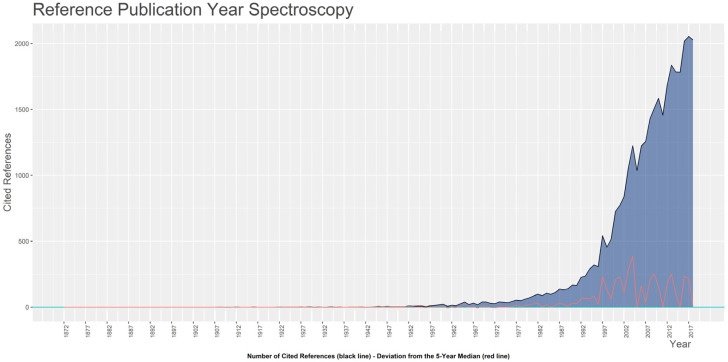
Spectrogram obtained from the analysis of cited references by the authors using the “Reference Publication Year Spectroscopy” (RPYS) method [51]. The black line represents the number of citations obtained for each reference publication year (RPY) of PLF documents in the collection, while the red line shows the deviation from the running median with a 5-year window for each RPY.

The interplay between current research and past literature plays a significant role in understanding which ideas, theories, or studies have influenced and outlined the routes of PLF research. The analysis of cited references by the authors can be used to answer these and related questions. In this context, we applied the “Reference Publication Year Spectroscopy” (RPYS) method, introduced by Marx [51], to investigate the historical roots of research fields. RPYS is a quantitative method based on analyzing the frequency of cited references in a specific field according to the publication year. Figure 4 shows the spectrogram, which displays the number of citations obtained for each RPY in the timespan considered and the 5-year median deviations.

Under the curve, certain RPYs appear particularly frequent in the references and are displayed as distinct peaks in the curve. By analyzing the publications underlying the curve, it is possible to identify the individual highly cited publications by the authors in the collection for each RPY, which are presented in Table 3. Full details are provided in Table S6, where documents cited at least 10 times locally are temporally ordered. These highly cited references should be considered the most impactful publications for advancing knowledge in this sector and reviewed for their significance [51].

**Figure 5:**
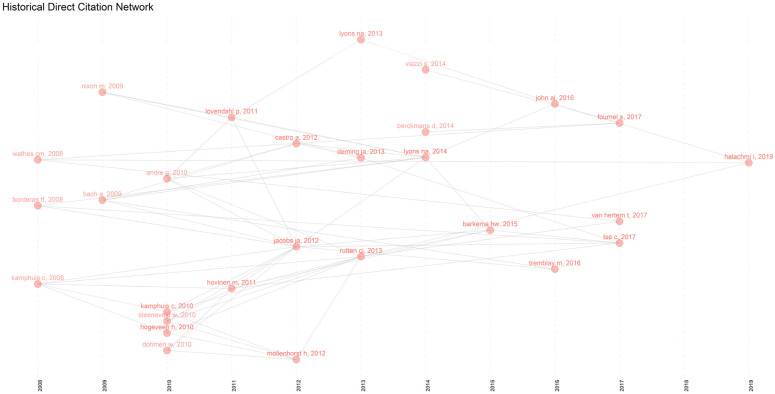
Chronological map of the 30 most relevant citations resulting from the PLF bibliographic collection using Garfield’s method of historiography [61]. References are present in Table 4 [16,19,20,21,22,23,27,29,33,36,39,40,41,42,43,44,45,46,47,48,49,50,62,63,64,65,66,67,68,69].

Parallel to RPYS, we also applied Garfield’s method of historiography to study the role of past literature in improving current knowledge [61]. This method creates a time-based network of key scientific publications, identifying their chronology and impacts. Each node in the network represents a key event (Figure 5; Table 4) that served as the basis for further research development in the field. Our results demonstrate the evolution of key events that influenced PLF growth and progress. Our perspective highlights the role of automation during milking procedures as a primary driver of the dissemination of technological innovations in the PLF sector [20,46,47,49,62] (Table 4). The health and welfare of animals in intensive livestock systems were also driving forces in PLF research [19,21].

For instance, papers that highlighted the importance of combining in-line composite somatic cell count (ISCC) and electrical conductivity (EC) information to improve the performance of a clinical mastitis (CM) detection system during automatic milking, as well as the validation of data mining techniques as CM detection models to reduce the number of false-positive alerts, have made influential contributions to the studies that followed [44,48,63]. Additionally, the arrival of real-time monitoring of animals by sensors and new management systems capable of improving animal well-being by providing warnings when something goes wrong was certainly a key factor that changed the way animals were managed, as evidenced by studies such as [23,33,64].

#### 3.1.5. Document Co-Citation Analysis: Semantic Similarity

**Figure 6:**
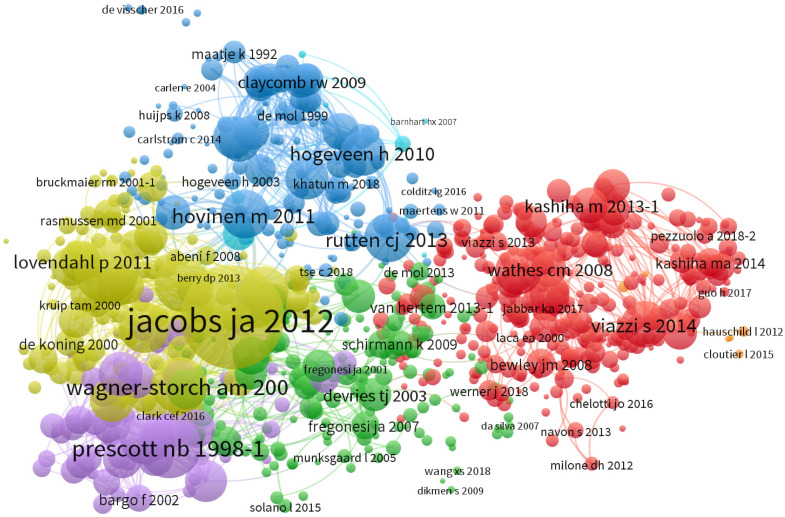
Reference co-citation network generated by VOSviewer. Magnitude of number of citations is indicated by font. All references are listed in Supplementary Table S7.

A co-citation relationship exists when two documents are cited together. Co-citation clustering analysis was performed on the documents to understand the connections between the most-cited articles and to identify potential thematic clusters and research directions. The result was a co-citation network in which all documents in the collection were analyzed and clustered into seven groups, as shown in Figure 6 and in Supplementary Table S7. Each cluster was represented by papers that were highly cited together with other documents; therefore, these publications belong to the same group due to the semantic similarity of the topics. The first four clusters were more representative and are discussed here because they contained the largest numbers of publications.

The red cluster of co-citations is considerably larger than the other groups, consisting of 344 documents. Fully automatic monitoring enables the evaluation of animal behavior, which is considered a crucial element in defining animal welfare [23,70]. The concept of developing and utilizing automatic image processing techniques in livestock production to monitor animal welfare is not new, and several studies have been conducted on this issue since the 1990s [71]. The common theme of the red cluster appears to be digital image processing for behavior monitoring and problem detection [36] and estimating body condition scores or weight [72,73,74,75]. In fact, image analysis can assist farmers in automatically identifying pigs [76] and their lying behavior [77] and in enhancing pig welfare, since issues with access to water or abnormal drinking behavior can be reported before the manifestation of health problems [78]. The early detection of diseases, such as lameness in dairy cows [79,80,81], and the measurement of individual cow feed intake are other important applications of these technologies [64]. Additionally, on the periphery of the red cluster, there are some documents based on modern techniques of sound analysis. The analysis of vocalizations has also been considered a promising tool for understanding the health state and welfare of animals because specific behaviors are often accompanied by particular sounds that can be recorded and interpreted [82,83,84,85].

The green cluster, consisting of 162 documents, is slightly smaller than the red group but occupies a central position with strong connections to both the red and yellow clusters. The co-citation analysis for this cluster highlights how the automation-related issues are connected with those regarding the impact of the automation technologies on cattle behavior. This is particularly relevant, as automation can affect feeding and lying timing throughout the day, as well as voluntary milking behavior in the presence of AMSs [50]. The paper by Barkema et al. [19] underlined the tremendous potential of many of these technologies to improve health, welfare, and reproductive performance. It also reported specific cases, such as the introduction of an automated calf feeder for calf health and the use of AMSs to provide data supporting the prevention of health problems like mastitis. The AMS represents the most important innovation with a great interdependence with behavior. Therefore, the green cluster confirms that concerns related to AMS introduction and management were associated with a renewed interest in understanding cattle behavior. Among the interrelated topics, feeding behavior [86] and cow mobility (i.e., lameness incidence) were the most important for successful AMS management [16,87,88].

The yellow cluster is highly related to the first two clusters and consists of 140 documents. It represents an important group of papers focusing on AMSs and their related technologies. As mentioned earlier in this paper, the development and introduction of milking robots have been a significant driving force for the advancement of new technologies on farms [56,89]. Many published studies on AMSs have focused on animal welfare, behavior, herd management, as well as the potential impact on milk production [90,91,92], milk quality [93], and various metabolic aspects [94]. In the yellow cluster, the paper by Jacobs and Siegford [20] is a central reference for the transition from the first phase of PLF development, which was strongly linked to the spread of AMSs and their associated technology, to the application of new sensors on cows and conventional parlor milking systems. The most common theme in this cluster is the relationship between AMS management and cow health, which affects milk quality and is reflected in metabolic and infection aspects.

Detecting mastitis is an important aspect of health management in dairy farms [95], and the blue cluster (consisting of 150 documents) mainly includes documents related to mastitis and detection sensor systems [96]. Mastitis has a significant economic impact [97], which is why sensor systems for detecting this condition in dairy cows are significant thematic goals of PLF to support udder health management [21,27,29,52,98,99,100]. Early research mainly focused on the electrical conductivity (EC) of milk, as this required a technology that was simple and cost-effective to implement [101], and it became the most-studied sensor system [52,98,102]. Interestingly, farmers’ opinions on mastitis detection systems showed a preference for high-specificity systems that produce a low number of false alerts, provide timely warnings, and pay particular attention to the most serious cases [65].

Likewise, a journal co-citation network was constructed to examine the influence of the PLF topic on the source journals (Table S8). The analysis grouped the journals into three main clusters and provided evidence, also at the journal level, for the three pillars of PLF scientific development. While the *Journal of Dairy Science* is the leading journal in dairy sciences, *Applied Animal Behavior Science* suggests that a significant share of research for sensor applications derived from a great increase in the applied ethology field (time budget, feeding behavior, etc.). The presence of *Computers and Electronics in Agriculture* was obvious, considering the leading role of this journal in publishing papers on digital technologies in agriculture.

#### 3.1.6. International Research Collaboration

**Figure 7:**
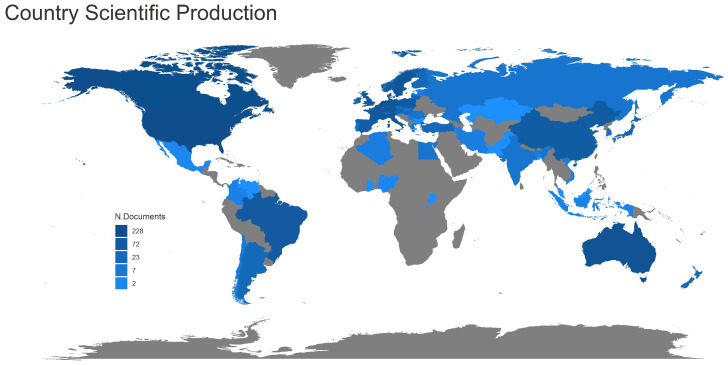
Worldwide scientific production of PLF-related publications by country affiliations, measured by the number of author appearances.

Considered the geographical distribution of publications, researchers from 58 different countries participated in producing all the collected documents (Table S9). When examining top-ranking countries, the analysis gives higher ratings to several English-speaking and European countries, such as the USA, Canada, the Netherlands, Australia, Italy, Belgium, Germany, Denmark, Sweden, the UK, and France, as shown in Figure 7.

**Figure 8:**
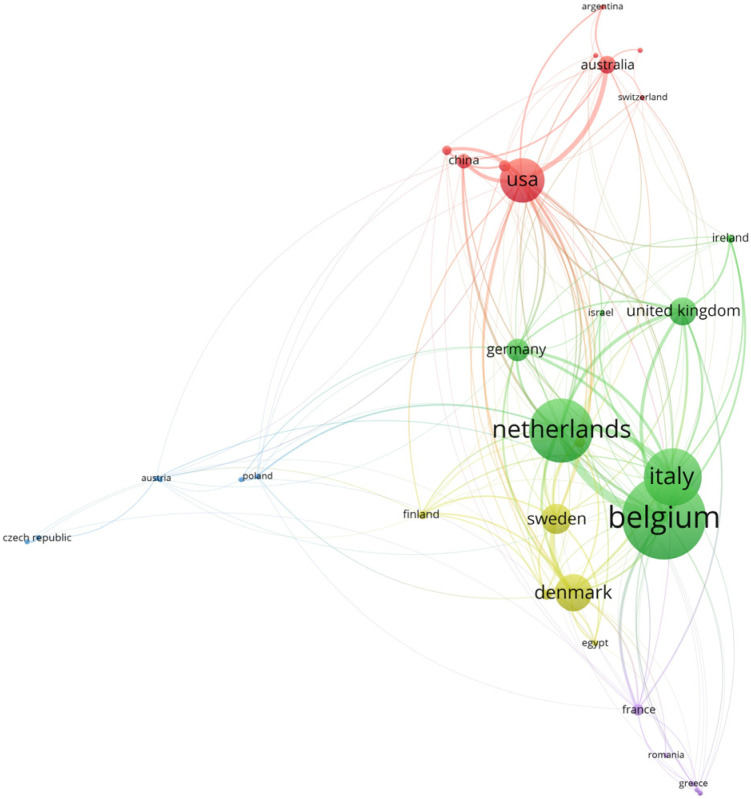
Network visualization map of international research collaboration in the field of PLF generated by VOSviewer. Countries that are closer to each other and connected by thick lines indicate strong research collaboration, whereas countries located on the periphery with thin connecting lines to countries in the center indicate limited international research collaboration. The font size represents the number of papers published in collaboration with another country, indicating the magnitude of collaboration.

In terms of corresponding author affiliations, the USA had the highest number of published articles (83 articles), followed by the Netherlands (74 articles), Italy (68 articles), Australia (66 articles), and Canada (53 articles) (Table S10). The rate of cooperation between countries is shown in Figure 8, based on networking activity among researchers studying PLF-related topics. In the map, some countries were located on the margin of the network with thin connecting lines to the countries in the center, indicating poor collaboration between countries at the periphery and those in the center. Network analysis points out that European researchers collaborate preferentially with each other, with a strong collaboration between the Netherlands, Italy, and Belgium. The country collaboration world map is also shown in Figure S3.

The multiple country publication ratio (MCP_Ratio) of the collection, based on the number of documents produced by researchers affiliated with more than one country, was approximately 29% (USA, MCP_Ratio 35%; Netherlands, MCP_Ratio 23%; Italy, MCP_Ratio 23%; Australia, MCP_Ratio 17%; and Canada, MCP_Ratio 21%) (Table S9).

### 3.2. Text Mining Analysis

#### Most Frequent Words: Author Keywords and Keywords Plus

**Figure 9:**
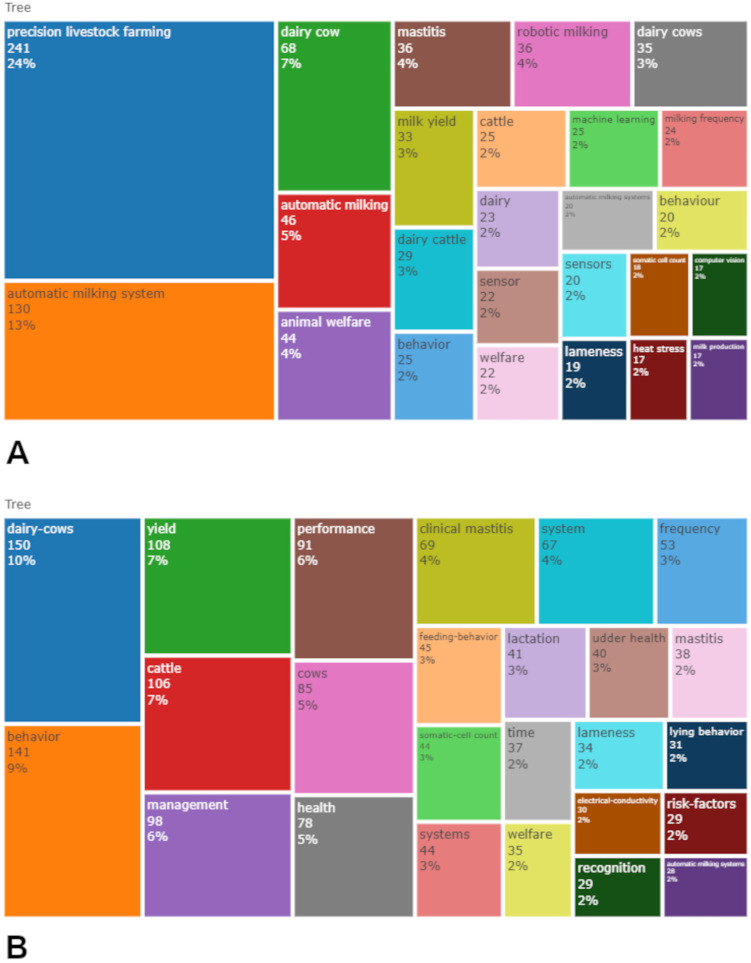
TreeMaps generated by the WoS. (**A**) TreeMap visualization of the top 25 author keywords in the PLF bibliographic collection. (**B**) TreeMap visualization of the top 25 Keywords Plus in the PLF bibliographic collection.

The total number of author keywords was 1868, with “precision livestock farming”, “automatic milking system”, “dairy cow”, “animal welfare”, “mastitis”, “milk yield”, “behaviour”, and “machine learning” being the most commonly used. The top 25 author keywords are shown in the TreeMap of Figure 9A. Keywords Plus, which are words that frequently appear in the titles of cited publications and are obtained by the algorithm of the WoS, were also analyzed to further understand article contents [103]. The total number of entries retrieved was 1560. The most frequent Keywords Plus are shown in the TreeMap of Figure 9B.

The comparison of author keywords and Keywords Plus showed that the leading term in PLF development (at least from the point of view of the scientific literature) is “dairy cow”. It was immediately clear that most PLF-related papers are dominated by Keywords Plus related to dairy cows and that most other terms are directly linked to these, such as “behavior”, “yield”, “cattle”, “management”, “feeding behavior”, “health”, “welfare”, “clinical mastitis”, “lameness”, etc. All of these items have already been identified and highlighted in the present paper, confirming our results.

**Figure 10:**
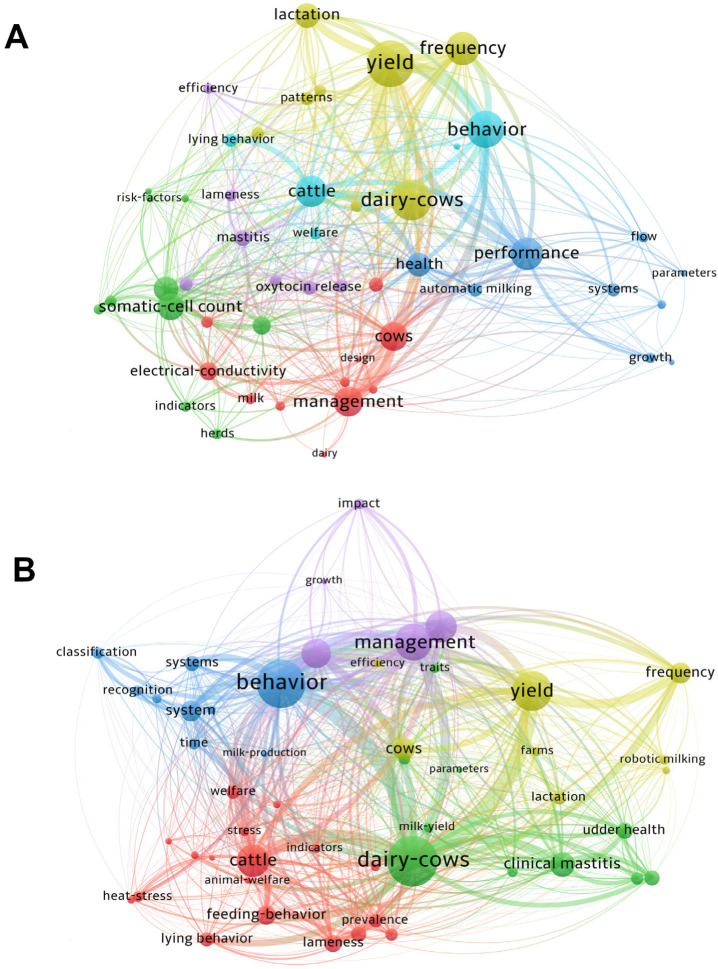
Co-occurrence network analysis of the top 50 Keywords Plus generated by VOSviewer. The Keywords Plus are organized into clusters (minimum cluster size: five items), and the strength of their relationship is represented by the thickness of the connecting lines. The size of each node corresponds to the frequency of the respective Keyword Plus. Panels (**A**,**B**) depict the co-occurrence networks for the periods 2008–2015 and 2016–2021, respectively.

The co-occurrence network analysis, which visualizes the relationships between the 50 most frequent Keywords Plus in the collection, is shown in the maps of Figure 10A,B. The two networks were constructed by segmenting the entire collection into the periods 2008–2015 and 2016–2021, respectively, which are characteristic of the first and second phases with different annual growth rates of publication, as reported in Section 3.1.1. The aim was to visualize any transition of research issues in the past 13 years between the two periods and to capture the evolution of the topics in a more detailed way. The colours in Figure 10 represent the different clusters obtained by the analysis. The size of the circle node in the map is proportional to the frequency of the Keyword Plus. The distance and thickness of lines between words indicate the strength of their relationship. The co-occurrence network for Keywords Plus shows once again that “dairy cow” is at the core of PLF interests, as well as its growing link to the behavioural aspects of dairy cow management (Figure 10).

**Figure 11:**
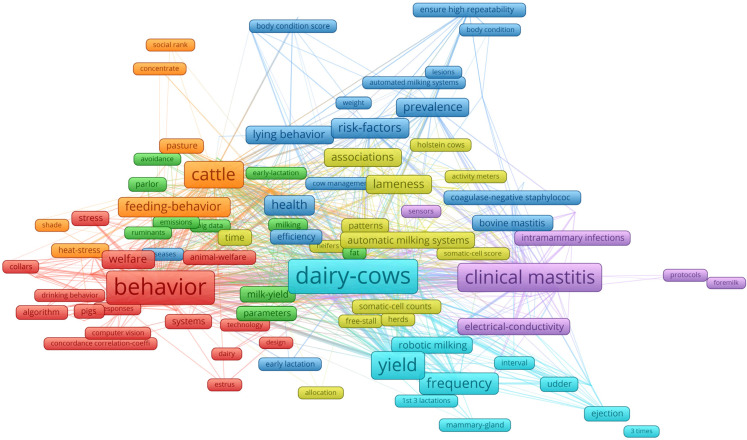
Co-occurrence network analysis (2008–2021) of the top 250 Keywords Plus derived from the keywords extracted from the 333 documents of core sources using Bradford’s law zones [18]. The network analysis was performed using VOSviewer. Each cluster in the network is represented by a distinct color, and a minimum cluster size of five items was applied for grouping the keywords.

Figure 11 shows the co-occurrence network analysis of the top 250 Keywords Plus retrieved by filtering the collection on the top more spread journals using Bradford’s law [18]. The results confirm that the main interests are related to “dairy cows” for the first cluster, followed by “behaviour” and “clinical mastitis”. This is not surprising, since clinical mastitis is the most costly pathology in dairy cows [104].

**Figure 12:**
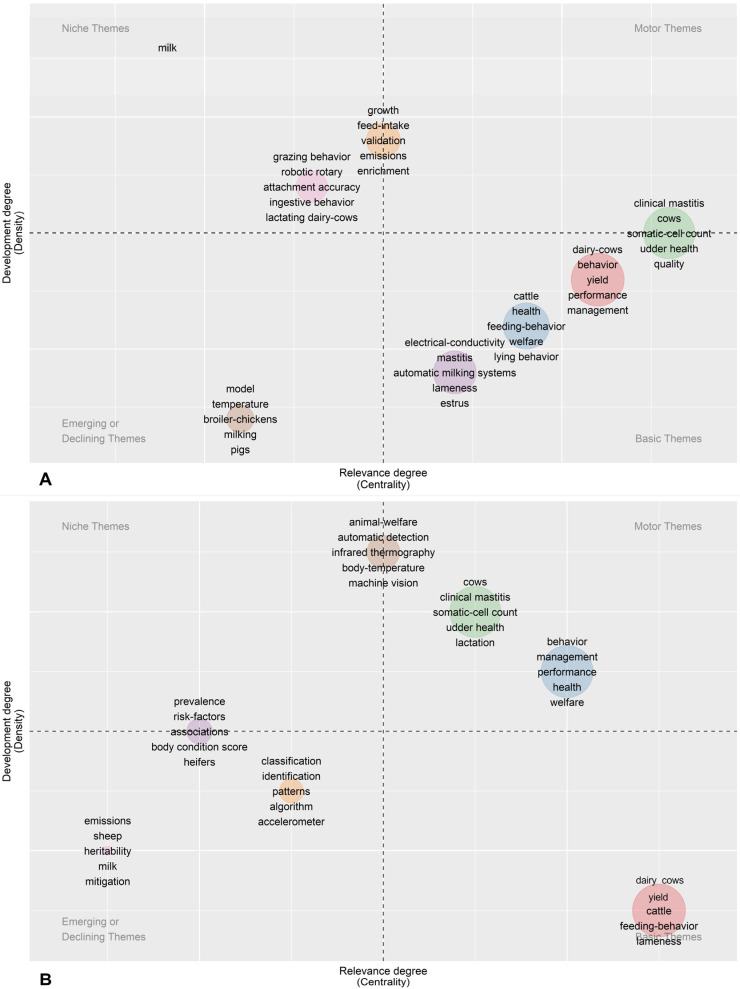
Illustration of the relevance of topics in PLF over time through thematic evolution. Two thematic maps were created using papers filtered by Bradford’s law [18] (Core and Zone2) for the periods 2008–2015 (**A**) and 2016–2021 (**B**). Each map shows clusters of the top 250 Keywords Plus (determined by co-word analysis through keyword co-occurrences) with the highest occurrence, which represent the most important topics in the field. The maps are characterized by measures of centrality and density and are divided into four areas. Centrality measures the relevance degree of a theme, while density shows the development of a theme.

Finally, Figure 12A,B illustrate the evolution of the PLF thematic in the core zone of the collection [18] during two periods, 2008–2015 and 2016–2021 [105]. The position of a bubble in the upper quadrants (y-axis) of the maps in Figure 12A,B is related to the degree of development or density, while the bubbles along the x-axis are related to the centrality or number of external links of a cluster. According to these indices, it is possible to divide the graph into four fields. The upper-left field is defined as niche topics. These are specialized topics characterized by strong internal relationships (high density) but weak external relationships (low centrality), and they have very little impact on the field of study. The upper-right field is defined as motor topics. These are highly developed and consolidated themes within the PLF world, as supported by high density and high centrality indices. The lower-right field is defined as basic topics. These are important topics in PLF, but their development is currently not active. The lower-left field is defined as emerging or disused topics. These topics may be new frontiers of PLF or fields of research already abandoned.

In our analysis, the first period (2008–2015) was not characterized by specific motor themes leading the scientific literature development. During the second period (2016–2021), jointly with the higher annual growth rate in scientific production, it was evident that the research on the applications of PLF to animal welfare, and specifically to high-economic-impact problems like mastitis in dairy cattle, was the leading field of development in the application of new technologies to livestock farming, joining applied behavior knowledge and management issues.

Among the emerging topics (lower left) in the second period, a growing interest in “emissions” and “mitigation” suggests how PLF is being applied in the management of environmental aspects related to livestock sciences. They will likely experience further growth in the next period, as there is growing attention on the possibility of PLF interacting with environmental impact aspects, for instance, through automation and mechatronics, as part of Agriculture 5.0 [106].

Among the basic themes in the first period were the aspects related to diagnostics in dairy cows (mastitis, lameness, and estrus).

## 4. Conclusions

The article presents a bibliometric analysis of the scientific literature on PLF, with a focus on dairy cattle management. It highlights the evolution of PLF technologies, from animal identification to the complete automation of farm processes through the implementation of artificial intelligence and machine learning. The bibliometric analysis sheds light on the leading sectors in livestock farming for PLF innovation, the technical areas expressing the greatest demand, and the countries leading the research, as well as collaboration networks and emerging research directions.

This study contributes to our understanding of PLF and its potential impact on the livestock industry. The findings suggest that there is an increasing interest in technology adoption in modern livestock farming, as reflected in the significant and consistent increase in PLF-related publications, especially after 2016. The countries with the highest numbers of publications were the United States, the Netherlands, Italy, Australia, and Canada. Moreover, the research collaboration network revealed strong collaboration among European countries.

The results obtained from the RPYS and historiography analyses of PLF research showed that automation during the milking procedure and the health and welfare of animals in intensive livestock systems were the driving forces behind PLF research. Overall, the RPYS and historiography analyses yielded insights into the historical origins and evolution of PLF research, which could serve as valuable guidance for future research in this field.

The results of co-citation clustering analysis of documents confirmed these findings and revealed four main thematic clusters of highly cited publications, showing a great interest in animal welfare and behaviour monitoring. The red cluster focused on the use of digital image processing to monitor animal behavior and detect problems automatically. The green cluster emphasized the interdependence between automation-related issues, feeding behaviour, lying, and voluntary milking behaviours. The yellow cluster was highly related to the first two clusters and focused on animal welfare, behaviour, and herd management within the context of automated milking systems. The blue cluster mainly referred to documents related to detection sensor systems and mastitis, an important aspect of dairy farm health management. Overall, these clusters represent distinct research themes related to automation and animal welfare in dairy farming.

The analysis of keywords confirmed that the dairy cow was the leading sector in PLF development, and the co-occurrence network analysis showed the growing link between the behavioural aspects of dairy cow management and PLF.

Finally, the study identified four fields classified as niche topics, motor topics, basic topics, and emerging topics that describe the evolution of the PLF thematic in the two periods (2008–2015 and 2016–2021). It is worth noting that the boundaries between these categories are not always clear-cut and that a topic may fall into more than one category, depending on the context. Additionally, the importance and relevance of these topics can change over time as new developments occur and our understanding of the world evolves.

Overall, the findings presented in this paper emphasize the need for further research, agricultural policy, and education to support the long wave of PLF innovation.

## Figures and Tables

**Table 1:** The top 10 of most frequent journals in the field of PLF.

Sources	No. of Articles	Percentage of Articles
*Journal of Dairy Science*	203	22.9
*Computers and Electronics in Agriculture*	76	8.5
*Animals*	54	6.1
*Animal*	35	3.9
*Biosystems Engineering*	29	3.3
*Livestock Science*	27	3.0
*Journal of Dairy Research*	20	2.2
*Journal of Animal Science*	19	2.1
*Animal Production Science*	15	1.7
*Sensors*	13	1.5

**Table 2:** (A). The top 20 globally most-cited papers in the field of PLF, based on data retrieved from the Web of Science Core Collection database. The papers are ranked in descending order according to the number of citations received as of the date of data retrieval. (B). The top 20 locally most-cited papers in the field of PLF, based on data retrieved from the Web of Science Core Collection database. The papers are ranked in descending order according to the number of local citations received as of the date of data retrieval.

**(A)**					
**Document**	**Title**	**DOI**	* TC	* TC_1_	* TC_2_
[19]	Invited review: Changes in the dairy industry affecting dairy cattle health and welfare	10.3168/jds.2015-9377	258	32.2	13.8
[21]	Invited review: Sensors to support health management on dairy farms	10.3168/jds.2012-6107	232	23.2	11.2
[22]	Is precision livestock farming an engineer’s daydream or nightmare, an animal’s friend or foe, and a farmer’s panacea or pitfall?	10.1016/j.compag.2008.05.005	162	10.8	5.5
[20]	Invited review: The impact of automatic milking systems on dairy cow management, behavior, health, and welfare	10.3168/jds.2011-4943	151	13.7	7.3
[23]	Precision livestock farming technologies for welfare management in intensive livestock systems	10.20506/rst.33.1.2273	124	13.8	5.8
[24]	Invited review: Effect of udder health management practices on herd somatic cell count	10.3168/jds.2010-3715	119	10.0	7.3
[25]	Systemic perspectives on scaling agricultural innovations. A review	10.1007/s13593-016-0380-z	110	15.7	5.6
[26]	Precision fish farming: A new framework to improve production in aquaculture	10.1016/j.biosystemseng.2017.10.014	102	20.4	11.1
[27]	Sensors and clinical mastitis—The quest for the perfect alert	10.3390/s100907991	87	6.7	3.7
[28]	Review: Environmental impact of livestock farming and precision livestock farming as a mitigation strategy	10.1016/j.scitotenv.2018.10.018	87	21.7	10.7
[29]	Invited review: Udder health of dairy cows in automatic milking	10.3168/jds.2010-3556	86	7.2	5.2
[30]	Accuracy of noninvasive breath methane measurements using Fourier transform infrared methods on individual cows	10.3168/jds.2011-4544	85	7.7	4.1
[31]	Near-infrared spectroscopic sensing system for on-line milk quality assessment in a milking robot	10.1016/j.compag.2008.01.006	77	5.1	2.6
[32]	Influence of milk yield, stage of lactation, and body condition on dairy cattle lying behaviour measured using an automated activity monitoring sensor	10.1017/S0022029909990227	76	5.8	3.3
[33]	General introduction to precision livestock farming	10.2527/af.2017.0102	76	12.7	4.6
[34]	Implementation of machine vision for detecting behaviour of cattle and pigs	10.1016/j.livsci.2017.05.014	75	12.5	4.5
[35]	Heritability estimates for enteric methane emissions from Holstein cattle measured using noninvasive methods	10.3168/jds.2015-10012	73	10.4	3.7
[36]	Image feature extraction for classification of aggressive interactions among pigs	10.1016/j.compag.2014.03.010	69	7.7	3.2
[37]	Recent advancement in biosensors technology for animal and livestock health management	10.1016/j.bios.2017.07.015	60	10	3.6
[38]	Development of automatic body condition scoring using a low-cost 3-dimensional Kinect camera	10.3168/jds.2015-10607	56	8	2.8
**(B)**					
**Document**	**Title**	**DOI**	* LC	* TC	* LC/TC
[20]	Invited review: The impact of automatic milking systems on dairy cow management, behavior, health, and welfare	10.3168/jds.2011-4943	91	151	60.26
[22]	Is precision livestock farming an engineer’s daydream or nightmare, an animal’s friend or foe, and a farmer’s panacea or pitfall?	10.1016/j.compag.2008.05.005	55	162	33.95
[23]	Precision livestock farming technologies for welfare management in intensive livestock systems	10.20506/rst.33.1.2273	51	124	41.13
[29]	Invited review: Udder health of dairy cows in automatic milking	10.3168/jds.2010-3556	45	86	52.33
[21]	Invited review: Sensors to support health management on dairy farms	10.3168/jds.2012-6107	45	232	19.40
[39]	Estimating efficiency in automatic milking systems	10.3168/jds.2010-3912	44	54	81.48
[33]	General introduction to precision livestock farming	10.2527/af.2017.0102	40	76	52.63
[19]	Invited review: Changes in the dairy industry affecting dairy cattle health and welfare	10.3168/jds.2015-9377	38	258	14.73
[40]	Covariance among milking frequency, milk yield, and milk composition from automatically milked cows	10.3168/jds.2010-3589	30	42	71.43
[41]	Comparison of 2 systems of pasture allocation on milking intervals and total daily milk yield of dairy cows in a pasture-based automatic milking system	10.3168/jds.2013-6716	29	30	96.67
[42]	Relationship between udder health and hygiene on farms with an automatic milking system	10.3168/jds.2009-3028	28	48	58.33
[27]	Sensors and clinical mastitis—The quest for the perfect alert	10.3390/s100907991	28	87	32.18
[43]	Factors associated with increased milk production for automatic milking systems	10.3168/jds.2015-10152	28	39	71.79
[44]	Detection of clinical mastitis with sensor data from automatic milking systems is improved by using decision-tree induction	10.3168/jds.2010-3228	26	45	57.78
[45]	Milking frequency management in pasture-based automatic milking systems: A review	10.1016/j.livsci.2013.11.011	26	33	78.79
[46]	Review: Milking robot utilization, a successful precision livestock farming evolution	10.1017/S1751731116000495	26	37	70.27
[47]	Forced traffic in automatic milking systems effectively reduces the need to get cows, but alters eating behavior and does not improve milk yield of dairy cattle	10.3168/jds.2008-1443	24	31	77.42
[48]	Automatic detection of clinical mastitis is improved by in-line monitoring of somatic cell count	10.3168/jds.2008-1160	23	50	46.00
[49]	Increasing the revenues from automatic milking by using individual variation in milking characteristics	10.3168/jds.2009-2373	23	32	71.88
[50]	Associations of housing, management, milking activity, and standing and lying behavior of dairy cows milked in automatic systems	10.3168/jds.2012-5985	23	46	50.00

* TC = total citations; * TC_1_ = total citations per year; * TC_2_ = normalized total citations. * LC = local citations; * LC/TC= local citations/total citations ratio (%).

**Table 3:** The most frequently cited references within the PLF collection for the relevant reference publication years that correspond to the peaks in Figure 4. Documents were ordered primarily by the running median with a 5-year window for each RPY (diffMedian5).

Year	* TCY	* diffMedian5	Document	Title	DOI	* LC
2004	1225	386	[52]	Electrical conductivity of milk: Ability to predict mastitis status	10.3168/jds.S0022-0302(04)73256-7	28
2003	1059	288	[53]	Feeding behavior, milking behavior, and milk yields of cows milked in a parlor versus an automatic milking system	10.3168/jds.S0022-0302(03)73735-7	37
2009	1509	253	[47]	Forced traffic in automatic milking systems effectively reduces the need to get cows, but alters eating behavior and does not improve milk yield of dairy cattle	10.3168/jds.2008-1443	24
2009	1509	253	[54]	Technical note: Validation of a system for monitoring rumination in dairy cows	10.3168/jds.2009-2361	23
2013	1836	252	[21]	Invited review: Sensors to support health management on dairy farms	10.3168/jds.2012-6107	45
2016	2019	235	[43]	Factors associated with increased milk production for automatic milking systems	10.3168/jds.2015-10152	28
1997	542	234	[55]	A review of livestock monitoring and the need for integrated systems	10.1016/S0168-1699(96)01301-4	19
2001	771	229	[56]	Sensors and clinical mastitis—The quest for the perfect alert	10.1016/S0301-6226(01)00276-7	50
2017	2053	217	[33]	General introduction to precision livestock farming	10.2527/af.2017.0102	40
2000	727	211	[57]	The effect of the introduction of automatic milking systems on milk quality	10.3168/jds.S0022-0302(00)75077-6	25
2008	1428	203	[22]	Is precision livestock farming an engineer’s daydream or nightmare, an animal’s friend or foe, and a farmer’s panacea or pitfall?	10.1016/j.compag.2008.05.005	55
2012	1685	176	[20]	Invited review: The impact of automatic milking systems on dairy cow management, behavior, health, and welfare	10.3168/jds.2011-4943	91
2006	1223	164	[58]	Cow traffic in relation to social rank and motivation of cows in an automatic milking system with control gates and an open waiting area	10.1016/j.applanim.2005.06.013	24
2010	1584	156	[42]	Relationship between udder health and hygiene on farms with an automatic milking system	10.3168/jds.2009-3028	28
2010	1584	156	[27]	Milking interval, milk production and milk flow-rate in an automatic milking system	10.3390/s100907991	28
1998	455	134	[59]	Relative motivations of dairy cows to be milked or fed in a Y-maze and an automatic milking system	10.1016/S0168-1591(97)00112-3	44
2002	839	112	[60]	Stress responses during milking; Comparing conventional and automatic milking in primiparous dairy cows	10.3168/jds.S0022-0302(02)74409-3	25
2014	1784	99	[23]	Precision livestock farming technologies for welfare management in intensive livestock systems	10.20506/rst.33.1.2273	51

* TCY = total citations per RPY; * LC = local citations per document; * diffMedian5 = deviation from the running median with a 5-year window for each RPY.

**Table 4:** Historiographic legend of most-cited papers ranked by year [61]. Each paper represents one of the nodes in Figure 5.

Document	Title	DOI	* LC	* TC
[16]	Effect of lameness on dairy cows’ visits to automatic milking systems	10.4141/CJAS07014	21	51
[22]	Is precision livestock farming an engineer’s daydream or nightmare, an animal’s friend or foe, and a farmer’s panacea or pitfall?	10.1016/j.compag.2008.05.005	55	162
[48]	Automatic detection of clinical mastitis is improved by in-line monitoring of somatic cell count	10.3168/jds.2008-1160	23	50
[62]	Genetic parameters of milking frequency and milk production traits in Canadian Holsteins milked by an automated milking system	10.3168/jds.2008-1689	20	35
[47]	Forced traffic in automatic milking systems effectively reduces the need to get cows, but alters eating behavior and does not improve milk yield of dairy cattle	10.3168/jds.2008-1443	24	31
[42]	Relationship between udder health and hygiene on farms with an automatic milking system	10.3168/jds.2009-3028	28	48
[49]	Increasing the revenues from automatic milking by using individual variation in milking characteristics	10.3168/jds.2009-2373	23	32
[27]	Milking interval, milk production and milk flow-rate in an automatic milking system	10.3390/s100907991	28	87
[63]	Discriminating between true-positive and false-positive clinical mastitis alerts from automatic milking systems	10.3168/jds.2009-3020	19	28
[44]	Detection of clinical mastitis with sensor data from automatic milking systems is improved by using decision-tree induction	10.3168/jds.2010-3228	26	45
[40]	Covariance among milking frequency, milk yield, and milk composition from automatically milked cows	10.3168/jds.2010-3589	30	42
[29]	Invited review: Udder health of dairy cows in automatic milking	10.3168/jds.2010-3556	45	86
[20]	Invited review: The impact of automatic milking systems on dairy cow management, behavior, health, and welfare	10.3168/jds.2011-4943	91	151
[39]	Estimating efficiency in automatic milking systems	10.3168/jds.2010-3912	44	54
[65]	Mastitis alert preferences of farmers milking with automatic milking systems	10.3168/jds.2011-4993	20	32
[66]	Comparing technical efficiency of farms with an automatic milking system and a conventional milking system	10.3168/jds.2012-5482	20	36
[41]	Comparison of 2 systems of pasture allocation on milking intervals and total daily milk yield of dairy cows in a pasture-based automatic milking system	10.3168/jds.2013-6716	29	30
[21]	Invited review: Sensors to support health management on dairy farms	10.3168/jds.2012-6107	45	232
[50]	Associations of housing, management, milking activity, and standing and lying behavior of dairy cows milked in automatic systems	10.3168/jds.2012-5985	23	46
[45]	Milking frequency management in pasture-based automatic milking systems: A review	10.1016/j.livsci.2013.11.011	26	33
[23]	Precision livestock farming technologies for welfare management in intensive livestock systems	10.20506/rst.33.1.2273	51	124
[36]	Image feature extraction for classification of aggressive interactions among pigs	10.1016/j.compag.2014.03.010	19	69
[19]	Invited review: Changes in the dairy industry affecting dairy cattle health and welfare	10.3168/jds.2015-9377	38	258
[46]	Review: Milking robot utilization, a successful precision livestock farming evolution	10.1017/S1751731116000495	26	37
[43]	Factors associated with increased milk production for automatic milking systems	10.3168/jds.2015-10152	28	39
[67]	Effect of transitioning to automatic milking systems on producers’ perceptions of farm management and cow health in the Canadian dairy industry	10.3168/jds.2016-11521	21	27
[68]	Rethinking environment control strategy of confined animal housing systems through precision livestock farming	10.1016/j.biosystemseng.2016.12.005	23	54
[69]	Appropriate data visualisation is key to precision livestock farming acceptance	10.1016/j.compag.2017.04.003	21	36
[33]	General introduction to precision livestock farming	10.2527/af.2017.0102	40	76
[64]	Smart animal agriculture: Application of real-time sensors to improve animal well-being and production.	10.1146/annurev-animal-020518-114851	19	52

* LC = local citations; * TC = total citations.

## Data Availability

The authors confirm that the data supporting the findings of this study are available within the article or the Supplementary Materials.

## Supplementary Materials

The following supporting information can be downloaded at: https://www.mdpi.com/article/10.3390/ani13142280/s1, Figure S1: Top authors’ productivity over time; Figure S2: Author local impact by H index; Figure S3: Country collaboration WorldMap; Table S1: Bibliometrix export file; Table S2: Main information about the collection; Table S3: Most relevant sources; Table S4: Most-cited sources; Table S5: Most relevant authors; Table S6: Reference publication year spectroscopy results; Table S7: Co-citation network of documents by WOSviewer; Table S8: Co-citation network of sources by WOSviewer; Table S9: Scientific productions per country; Table S10: Most relevant countries by corresponding authors.
