# Supplementary figures and images for: Scientific Productions on Precision Livestock Farming: An Overview of the Evolution and Current State of Research Based on a Bibliometric Analysis
Source: Animals (Basel). 2023 Jul 12;13(14):2280. doi: 10.3390/ani13142280 (PMC10376211; doi:10.3390/ani13142280)

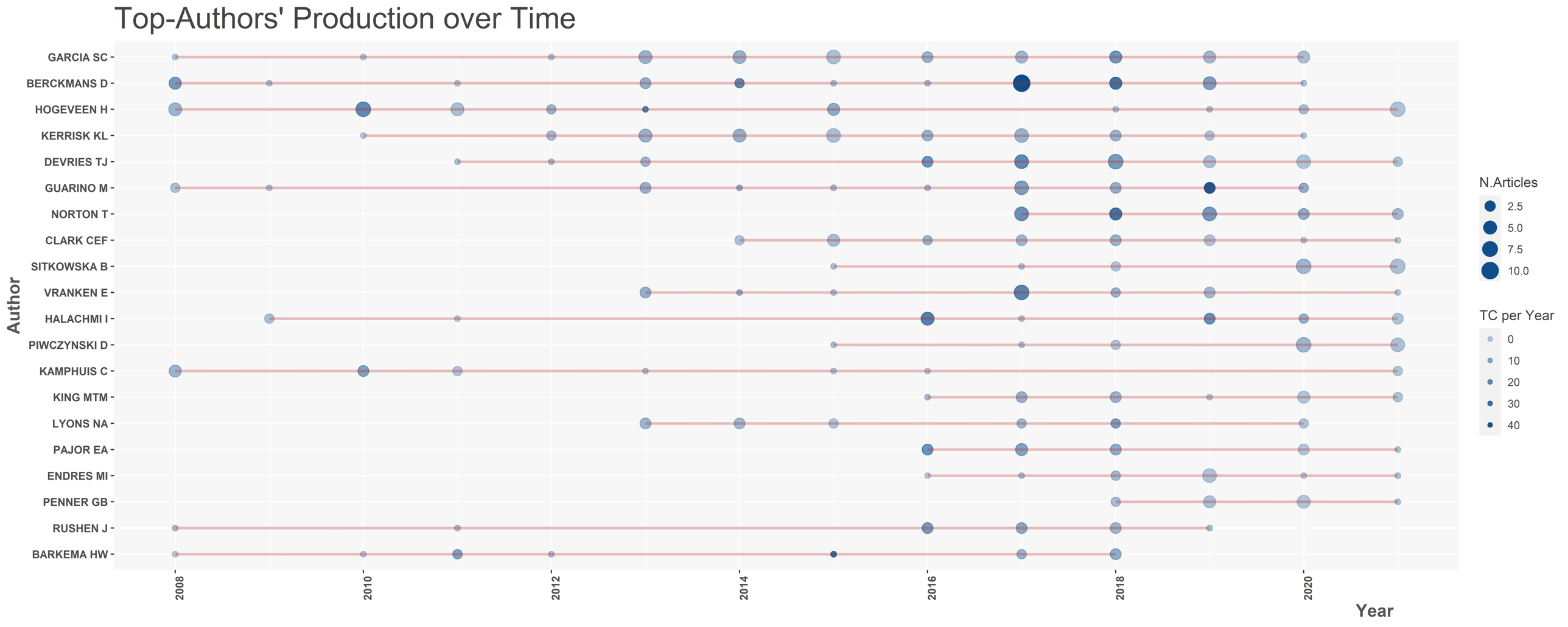

Supplement: Supplementary file 1 [file animals-13-02280-s001.zip › Figure S1 AuthorsProductionOverTime.jpg]

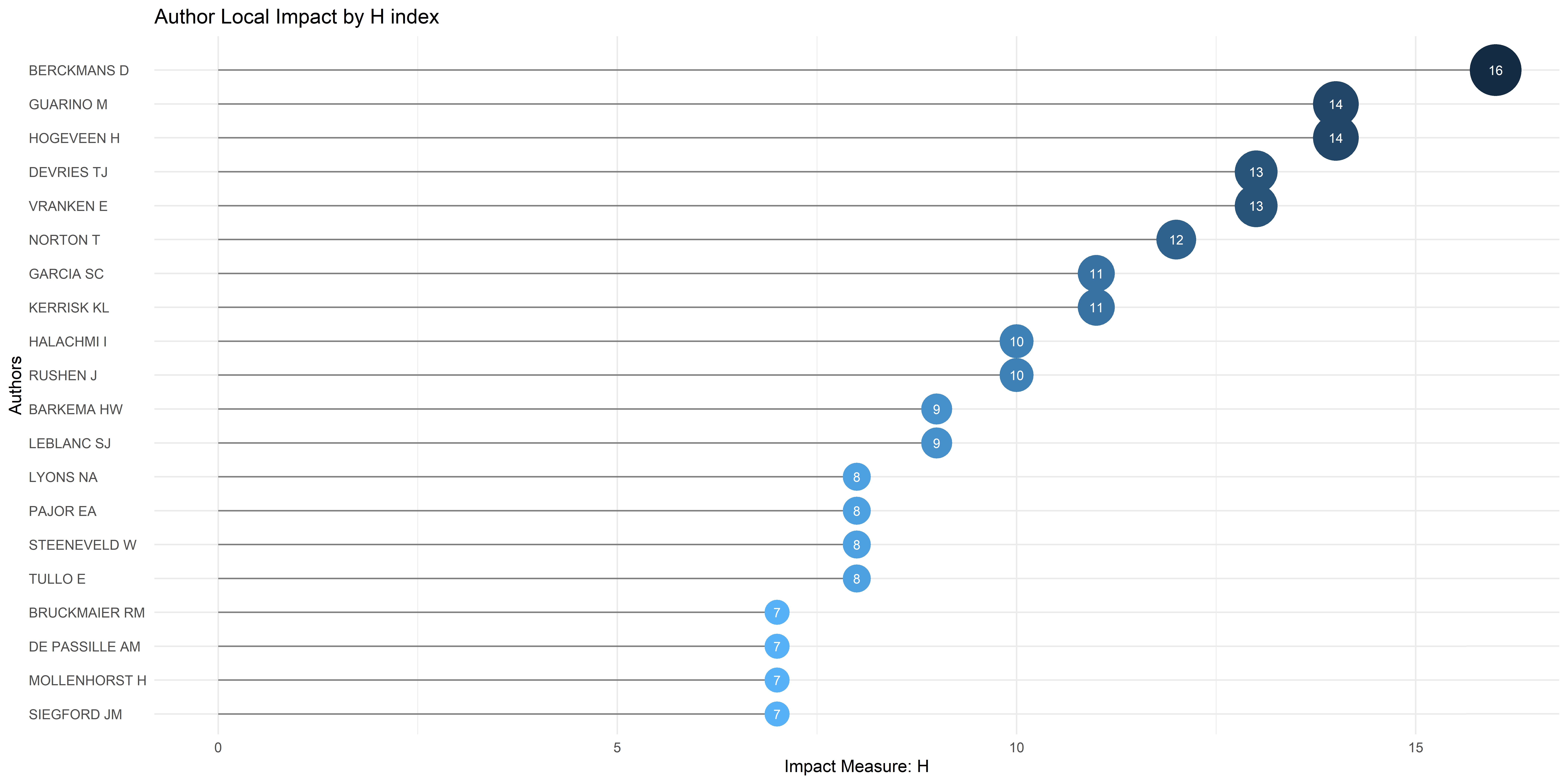

Supplement: Supplementary file 1 [file animals-13-02280-s001.zip › Figure S2 AuthorImpact.jpg]

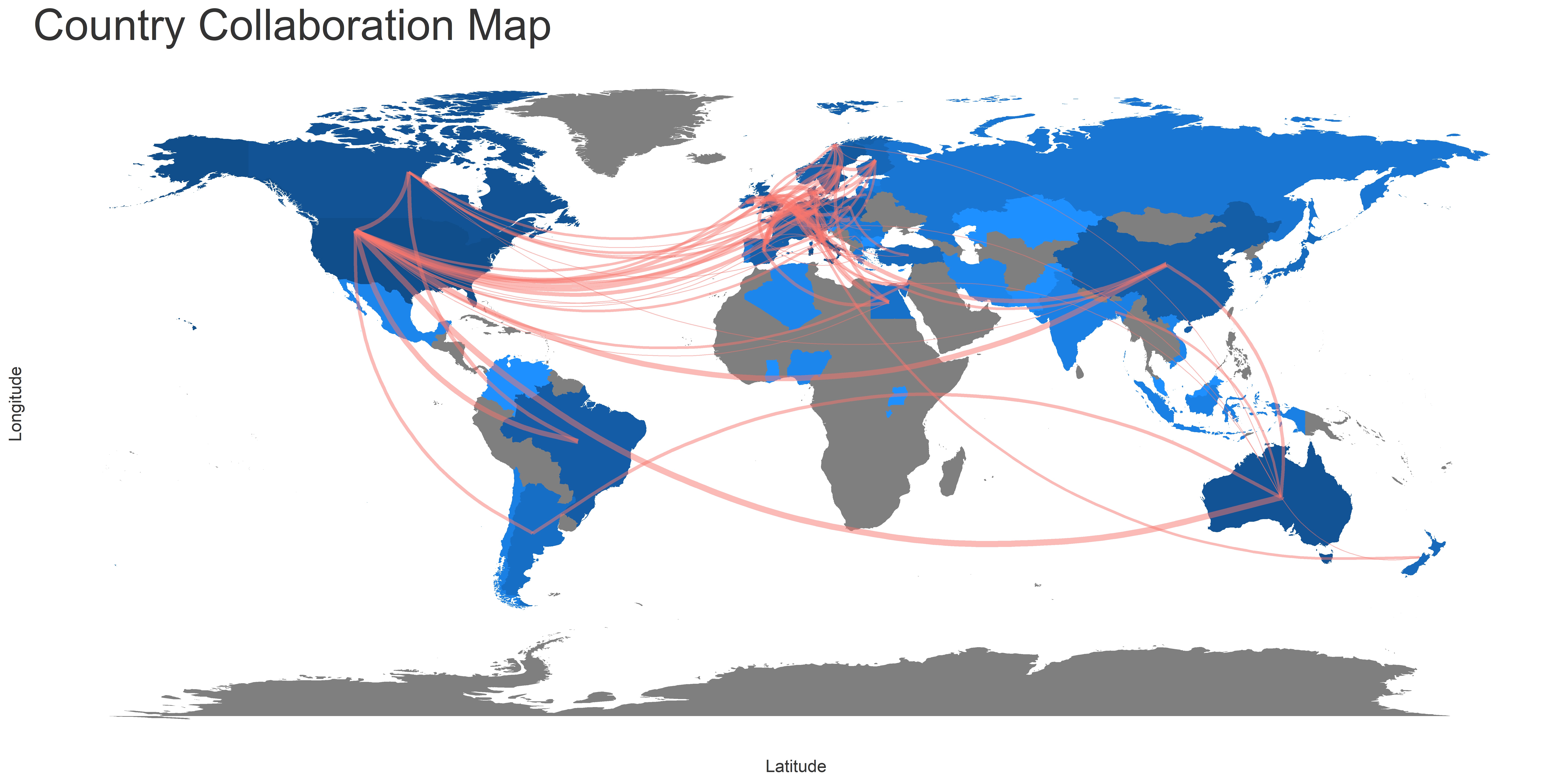

Supplement: Supplementary file 1 [file animals-13-02280-s001.zip › Figure S3 CountryCollaborationMap.jpg]
